# Spatial Distribution and Prognostic Value of T Cell Subtypes and Immune Biomarkers in p16-Negative HNSCC

**DOI:** 10.3390/cells14110789

**Published:** 2025-05-27

**Authors:** David Krum, Saskia Rösch, Rolf Warta, Carolin Mogler, Miray-Su Yılmaz Topçuoğlu, Niels Grabe, Patrick J. Schuler, Gerhard Dyckhoff, Christel Herold-Mende

**Affiliations:** 1 Department of Otorhinolaryngology, Head and Neck Surgery, Medical Faculty of Heidelberg, University of Heidelberg, INF 400, 69120 Heidelberg, Germany; david.krum@gnh.net (D.K.); rolf.warta@med.uni-heidelberg.de (R.W.); miray-su.yilmaztopcuoglu@med.uni-heidelberg.de (M.-S.Y.T.); patrick.schuler@med.uni-heidelberg.de (P.J.S.); gerhard.dyckhoff@med.uni-heidelberg.de (G.D.); 2 Division of Experimental Neurosurgery, Department of Neurosurgery, Medical Faculty of Heidelberg, University of Heidelberg, INF 400, 69120 Heidelberg, Germany; 3 Institute of Pathology, TUM School of Medicine, Technical University of Munich, Trogerstr. 18, 81675 Munich, Germany; carolin.mogler@tum.de; 4 Hamamatsu Tissue Imaging and Analysis Center, BioQuant, University of Heidelberg, INF 267, 69120 Heidelberg, Germany; niels.grabe@bioquant.uni-heidelberg.de

**Keywords:** HNSCC, TIL, survival, cytokine, regulatory T cell, CXCL10, IL-9, CCL4, VEGF

## Abstract

Patients with head and neck squamous cell carcinoma (HNSCC) suffer from severe morbidity and mortality. Immunotherapy represents a novel promising treatment option. Therefore, a better understanding of the immune niche is needed. This study focuses on the spatial distribution and prognostic value of different T cell subtypes in 84 HNSCC specimens as well as chemokine and cytokine levels associated with spatial T cell infiltration. Density of T helper (T_H_), cytotoxic (CTL), and regulatory T cells (T_reg_) was quantified by multicolor tissue cytometry on a single cell level in whole tissue sections, discriminating between T cells located in epithelial tumor cell nests or tumor stroma, respectively. In addition, quantitative levels of 27 immune-related factors were assessed. Survival analysis of patients with p16-negative HNSCC revealed higher stromal T_reg_ densities to be an independent prognostic factor for better progression-free and overall survival. Furthermore, high levels of CXCL10, IL-9, and CCL4 were associated with significantly higher numbers of T cells, especially for CTL with direct contact to tumor cells, whereas for VEGF the opposite effect was observed in the tumor stroma. In conclusion, T_reg_ cell infiltration as well as distinct cytokine levels could serve as new immune biomarkers in p16-negative HNSCC to predict survival and the spatial distribution of T cells.

## 1. Introduction

Head and neck squamous cell carcinoma (HNSCC) is responsible for more than 740,000 new cancer cases worldwide each year and more than 360,000 deaths [1]. The main risk factors are excessive consumption of alcohol and tobacco, and particularly the combination of both [2]. Furthermore, in the last decades, infections with human papilloma virus (HPV)—especially high-risk subtypes as HPV-16 or -18—have been causing rising numbers of new HNSCC cases [3]. Currently, HPV-associated HNSCC (mostly referred to as HPV positive or HPV^+^) and HNSCC that develops due to other risk factors (HPV negative, HPV^−^) are increasingly regarded as separate tumor entities due to substantial differences in pathogenesis, tumor biology, and patients’ prognosis.

As both HPV^+^ and HPV^−^ HNSCC arise from the mucosa of functionally highly relevant structures such as the oral cavity, the oro- and hypopharynx and the larynx, they lead to severe morbidity [4]. Treatment regimens are chosen based on the TNM status of the tumor and patients’ characteristics, such as comorbidities [5]. Standard therapy options are surgical resection of the tumor and lymph node metastases, radiation, chemotherapy, and immunotherapy with cetuximab or immune checkpoint inhibitors [6]. Most patients require combinations of these therapy options. Despite advances in recent years, the 5-year overall survival (OS) of patients suffering from HNSCC remains at about 50%, probably due to high rates of recurrence and the development of metastasis [7,8]. Overall, there is a strong need for new therapy options and biomarkers to guide treatment decisions. In recent years, many new anti-cancer therapies have been developed, including a number of immune-related therapies, with the aim to induce or improve anti-tumor immune responses [9]. Accordingly, a better understanding of the tumor immune microenvironment is of major importance. The immune microenvironment of HNSCC consists of many cell types including dendritic cells, macrophages, neutrophils, as well as stromal cells such as cancer-associated fibroblasts and endothelial cells [10]. However, a substantial number of immune cells in solid tumors and particularly in HNSCC are T cells (TC) due to the immunologically highly active mucosa [11]. Among them, activated CD8^+^ T cells differentiate to cytotoxic T cells (CTL), which have antimicrobial as well as anti-tumor effects, e.g., through apoptosis-inducing mechanisms such as perforin and granzyme secretion or Fas-ligand–Fas-receptor interactions [12,13]. 

A subgroup of CD4^+^ T cells support immune reactions through secretion of immune modulatory proteins and are called T helper cells (T_H_ cells). When activated, they can differentiate into different subtypes such as T_H_1 or T_H_2 cells based on surrounding cytokines. T_H_1 cells enhance antimicrobial and anti-tumoral immune reactions through secretion of Interleukin-2 (IL-2), tumor necrosis factor, and interferon-γ, leading to an enhanced function of antigen-presenting cells and enhanced cytotoxic activity of macrophages [13,14]. In contrast, T_H_2 cells reduce T cell mediated cytotoxic effects and promote B cell-mediated humoral immune reactions through secretion of IL-4, -5, -6, -10, and -13 [15]. T_reg_ represent another subtype of CD4^+^ T cells with strong immune modulatory functions. T_reg_ display a nuclear expression of forkhead box P3 (FoxP3) and can mediate immunosuppressive effects, e.g., through secretion of IL-10 and transforming growth factor-β [16,17].

Studies focusing on TC infiltration into HNSCC report conflicting results about the predictive value of different TC subtypes concerning patients’ survival [18]. However, previous studies have differed markedly regarding patients’ characteristics, methods to visualize, count, and evaluate TC infiltration, and regarding survival analyses [19]. These fundamental differences restrict the comparability and reproducibility of previous results [20]. Therefore, the aim of this work was to evaluate TC infiltration into HNSCC and its prognostic relevance as well as cytokine levels associated with T cell infiltration in a substantial patient cohort with an accurate and precise evaluation method to obtain reproducible and comparable results.

## 2. Materials and Methods

### 2.1. Patients and Tumor Samples

A total of 84 HNSCC specimens were obtained from patients at the Ear Nose Throat (ENT) Department of the University Hospital Heidelberg, Germany. All samples were obtained intraoperatively during primary tumor surgery before any adjuvant treatment. For the use of human tissue (tumor biopsies), ethics approval from the ethics committee at the University Hospital of Heidelberg was obtained (S-70/99, amendment 09/01/2004). Written informed consent was obtained from all patients involved in the study. Tumors were staged following the 7th edition of the UICC TNM classification, as it was the version used at the time of diagnosis and for treatment decisions. None of the patients had distant metastases at the time of diagnosis. Samples used for staining were immediately snap-frozen after surgery and stored at −80 °C until processing. Tumor cell content of at least 60% was confirmed for all samples by an experienced pathologist (C.M.). Clinical characteristics including survival data are summarized in Table 1.

### 2.2. Staining

**Figure 1:**
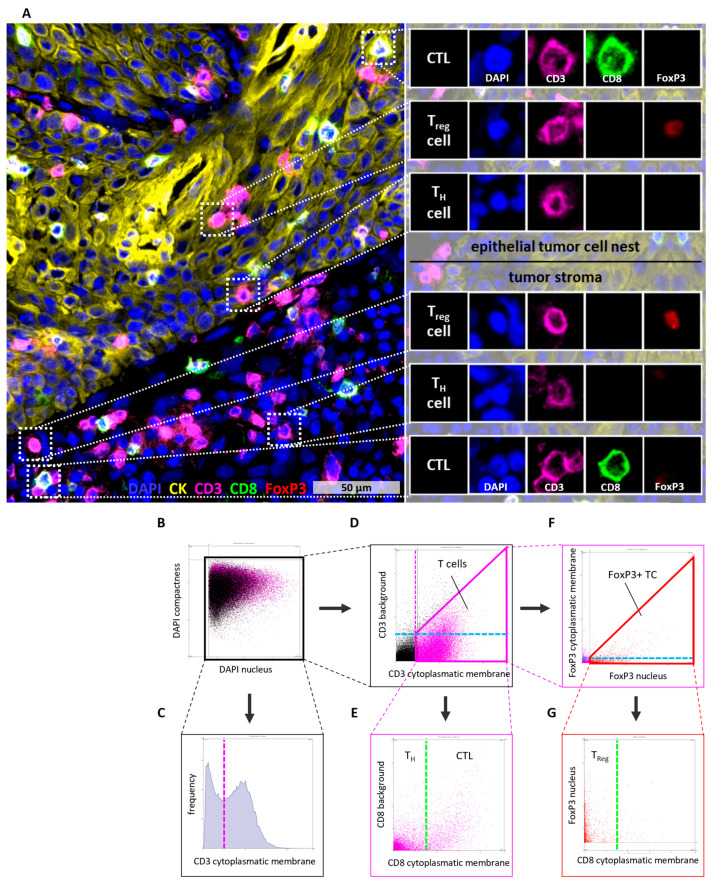
Multi color tissue cytometry for the detection of tumor and immune cells. (**A**) CD3, CD8, and FoxP3 were used as markers to classify T cells into cytotoxic T cells (CD3^+^CD8^+^FoxP3^−^), T helper cells (CD3^+^CD8^−^FoxP3^−^), and regulatory T_reg_ (CD3^+^CD8^−^FoxP3^+^). CK was used to determine epithelial tumor cell nests. (**B**–**G**) Gating algorithm in StrataQuest to determine T cell subsets. Gates are represented by solid lines, whereas dashed lines indicate the corresponding gate in a different graph. Consistent colors are used to denote equivalent gates or cutoffs. (**B**) Nuclei were detected based on the DAPI staining. (**C**,**D**) Detection of T cells was based on high CD3 cytoplasmatic/membrane staining and low CD3 background staining. These parameters were used to create a gate including all T cells. (**E**) To distinguish between T helper cells and cytotoxic T cells the CD8 cytoplasmatic/membrane staining was used. (**F**) FoxP3^+^ T cells were detected using high FoxP3 nuclear staining and low or no FoxP3 cytoplasmatic/membrane staining. These parameters were used to create a gate including all FoxP3^+^ T cells. (**G**) Finally, the number of T_reg_ (CD3^+^FoxP3^+^CD8^−^) was determined using the previous determined cutoff for CD8 cytoplasmatic/membrane staining. CK = Cytokeratin, CTL = cytotoxic T cell, DAPI = 4′,6-Diamidin-2-phenylindol, TC = T cell, T_H_ cell = T helper cell, T_reg_ = regulatory T cell.

To quantify T cell subpopulations, multicolor immunofluorescence staining (Figure 1A) was performed on acetone-fixed cryosections (4–6 µm) using a combination of primary antibodies specific for CD3 (rabbit, 1:100, A0452, Dako, Hamburg, Germany), CD8 (rat, 1:100, ab60076, Abcam, Cambridge, UK), and FoxP3 (mouse, 1:50, ab20034, Abcam). Staini, ng was performed as described previously [21]. Briefly, primary antibodies were diluted with Antibody Diluent (Dako) and incubated for 1 h. Secondary antibodies were used as follows: anti-rabbit AlexaFluor647 (1:200, Thermo Fisher Scientific, Waltham, MA, USA), anti-rat AlexaFluor488 (1:200, Thermo Fisher Scientific), and anti-mouse AlexaFluor555 (1:200, Thermo Fisher Scientific) for staining of CD3, CD8, and FoxP3, respectively. Secondary antibodies were diluted with DPBS-containing DAPI (1:1000, Invitrogen, Darmstadt, Germany) to stain nuclei and incubated for 30 min. Additionally, the cytokeratin (CK) antibody AE1/AE3 (mouse, 61835, Progen, Heidelberg, Germany) was first conjugated with PerCP, following the manufacturer’s instructions (PerCP Conjugation Kit, Abcam) and then used for staining (dilution 1:41). PerCP conjugated CK antibodies were incubated for 1 h.

To determine the p16 status, immunohistochemical staining was performed. The primary antibody specific for p16 (mouse, 1:50, 550834, BD Pharmingen, Heidelberg, Germany) and an isotype control (human, 1:160, ab91353, Abcam) were diluted with Antibody Diluent (Dako) and incubated for 45 min at 37 °C. Primary antibody binding was detected using the Vectastain Elite ABC Kit (HRP) (mouse, Vector Laboratories, Burlingame, CA, USA) according to the manufacturer’s instructions and the enzymatic staining reaction was stopped after 30 min. Positive staining was classified according to a positive control (tissue with known p16^+^ staining). Nuclei were counterstained using hematoxylin.

### 2.3. Image Analysis

High-resolution whole slide scans of tissue sections were acquired using a 20× objective on an Olympus IX51 microscope equipped with a XM10 camera (Olympus, Hamburg, Germany). The Olympus CellSens Dimension software (version 1.9) was used for image acquisition. Semi-automated detection and quantification of T cell infiltration by immunofluorescence staining was performed by the StrataQuest software (version 5.0.1, TissueGnostics GmbH, Vienna, Austria) according to a defined algorithm (Figure 1B–G).

To ensure a high quality of the analyzed area, necrotic regions were excluded based on CK staining as well as by a separate hematoxylin and eosin staining. Detected cells were visualized in scattergrams and gated according to defined gating schemes for the expression of nucleic and cell surface markers (Figure 1B–G). The cutoff for determining marker-positive cells was validated by backward gating.

Stringent parameters, including cell nucleus size, staining intensity, and background threshold, were defined to ensure robust and reliable cell quantification. Cell nuclei were detected based on DAPI staining and used to generate a growing mask across the cytoplasm to the cell membrane. Based on this mask, T cells were analyzed regarding expression of CD3 and CD8 and a nuclear colocalization of FoxP3 and DAPI (Figure 1). To distinguish between epithelial tumor cell nests and tumor stromal localization of cells, CK staining was used to create a distance transformation layer, depicting, for every pixel, the distance to the nearest epithelial tumor area as a continuous increasing intensity. After manual correction to exclude areas with unspecific CK staining, the distance of each T cell was calculated based on these retrieved intensity values (Supplementary Figure S1).

### 2.4. Luminex Assay

For further analysis of the immunologic micromilieu, cytokine and chemokine concentrations of 40 p16^−^ tumor samples were measured. First, additional tumor tissues undergoing the same quality control with regard to the tumor cell content were lysed and their protein concentrations were measured and normalized to 1 mg/mL. Then, a Luminex assay was performed for the quantification of the cytokines and chemokines.

For the lysis of tumor tissues the Bio-Plex Cell Lysis Kit (Bio-Rad Laboratories, Hercules, CA, USA) was used. All steps of the protein extraction were performed at 4 °C and Protein LoBind Tubes (Eppendorf, Hamburg, Germany) were used.

A Human Cytokine 27-Plex Assay Kit (Bio-Rad Laboratories, Hercules, CA, USA) was used for the quantitative analysis of 27 cytokines and chemokines in the tumor lysates, following the instructions of the manufacturer. This panel represents a biologically relevant collection of cytokines involved in adaptive immunity cytokines, pro-inflammatory cytokines, and pro- and anti-inflammatory cytokines. First, the protein concentrations of all tumor lysates were equalized to 1 mg/mL using the kit’s sample diluent. Then cytokine-specific antibodies bound to fluorescence-marked magnetic beads detected via red and infrared light were added. To quantify the cytokine concentrations, biotinylated antibodies bound to streptavidin and conjugated with phycoerythrin were used. All steps were performed in duplicate and a standard dilution series with known cytokine concentrations was used as a reference. The readout was performed using the Bio-Plex Reader and the cytokine concentrations were calculated automatically by the software Bio-Plex Manager (version 6.1) based on fluorescence intensities.

### 2.5. Statistical Evaluation and Visualization

Data visualization was performed using the software GraphPad Prism (9.0.0). Univariate and multivariate survival analyses were performed using the software R (4.4.1). Differences in T cell infiltration parameters were analyzed using Mann–,Whitney *U* Test. For the comparison of intra-tumoral and intra-stromal T cell infiltration the Wilcoxon matched-pairs signed rank test was used. For visualization, boxplots with median, interquartile range, and whiskers from minimum to maximum were used.

Survival data were retrieved from patient files. Overall survival and progression-free survival were defined as the time from beginning of treatment until death or tumor progression, respectively. Univariate survival data were visualized as Kaplan–Meier curves with *p*-values based on log rank test, while a Cox proportional hazard model (PH) was used to conduct multivariate survival analysis. When necessary, continuous variables were split into two groups based on the median. Missing values of the cytokine detection assay (1.7%) as well as three outliers (5× standard deviation) were excluded from correlation analysis. Pairwise datasets of protein concentrations and T cell infiltration of the same tumor tissue were analyzed using the corrplot package in the software R to calculate and visualize Spearman correlations.

## 3. Results

### 3.1. Patient Characteristics

To analyze the spatial distribution of distinct T cell phenotypes and their impact on patient survival, a study collective of 84 patients suffering from HNSCC was used. In addition, in a subset of 40 p16^neg^ patients, chemokine and cytokine expression levels were quantified. A summary of patient characteristics is shown in Table 1.

All tumors were collected at the time of first surgery, before adjuvant treatment and thus can be regarded as therapy-naive. Similar numbers of tumors of major anatomical sites, such as oro- and hypopharynx, oral cavity, and larynx, were included. The male-to-female ratio was approximately 4.7:1, with a median age of 60 years. These values are in line with the average data for HNSCC patients in Germany [22]. The most common UICC stage among the patients was stage IVa, with tissues from all four T stages and N stages 0, 1, and 2 included. Postoperative therapy was administered to 81% of patients including radiation alone (42.9%) and combined chemoradiotherapy (38.1%).

### 3.2. Tumor-Infiltrating Lymphocytes

The infiltration of lymphocytes into the tumor tissue was assessed using the markers CD3, CD8, and FoxP3. Based on these antigens, T cells were subdivided into general CD3^+^ T cells, T helper cells (T_H_ cells, CD3^+^CD8^−^FoxP3^−^), cytotoxic T cells (CTL, CD3^+^CD8^+^FoxP3^−^), and regulatory T cells (T_reg_, CD3^+^CD8^−^FoxP3^+^, Figure 1). The main parameter to describe T cell infiltration was cell density (cells/mm^2^). As a surrogate marker for tumors resulting from an infection with human papilloma virus (HPV), the p16 status of the tissues was determined (Table 1).

### 3.3. Differences in T Cell Infiltration Depending on p16 Status

**Figure 2:**
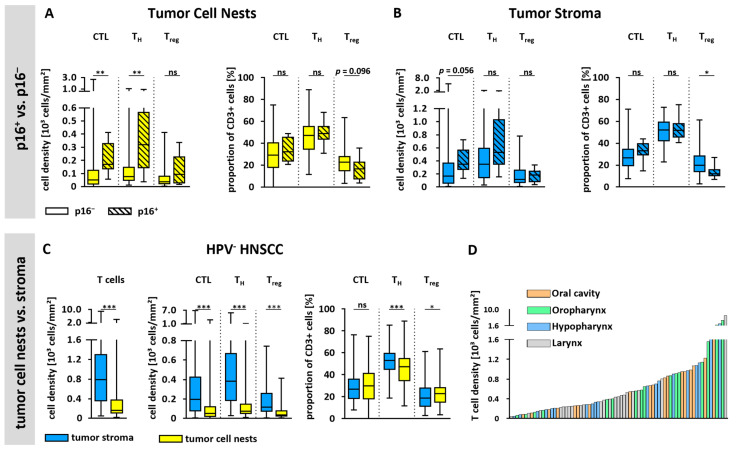
T cell infiltration based on p16 status, spatial distribution, and anatomical site. (**A**,**B**) T cell infiltration of p16^+^ (n = 8) and p16^−^ (n = 76) tissues within (**A**) epithelial tumor cell nests and (**B**) tumor stroma. T cell densities of CTL, T_H_ cells, and T_reg_ as well as proportions within the whole T cell population are shown. (**C**) Densities of all T cell subsets and their proportions are shown based on spatial distribution in tumor cell nests and tumor stroma of p16^−^ tumors. (**D**) Bar plot showing all p16^−^ tumors (n = 76) ordered by T cell density in entire tissue and colored based on the anatomical tumor site. Statistical test was unpaired Mann–Whitney *U* Test in (**A**,**B**) and paired Mann–Whitney *U* Test in C. ns = not significant; * *p* < 0.05; ** *p* < 0.01, *** *p* < 0.001.

There were substantial differences in the overall T cell infiltration between p16 negative (p16^−^, n = 76) and p16 positive (p16^+^, n = 8) HNSCC (Figure 2A,B). In the tumor cell nests, median CTL density as well as median T_H_ cell density were higher in p16^+^ tissues than in p16^−^ tissues (169 CTL/mm^2^ vs. 52 CTL/mm^2^; 318 T_H_ cells/mm^2^ vs. 75 T_H_ cells/mm^2^). However, instead of a similar trend, there were no significant differences regarding T_reg_ densities in the tumor cell nests depending on p16 status.

In the tumor stroma, overall median T cell densities were also higher in p16^+^ tissues (Figure 2B). However, these differences did not reach statistical significance, perhaps due to the small number of p16^+^ tissues. Furthermore, the proportions of CTL and T_H_ cells showed no differences depending on p16 status in either tumor cell nests or tumor stroma. In contrast, the proportion of T_reg_ was significantly lower within the tumor stroma of p16^+^ tissues (16.7% vs. 20.1%, *p* = 0.031) and showed a tendency to be lower in the tumor cell nests of p16^+^ tissues. Overall, due to the substantial differences between p16^+^ and p16^−^ HNSCC, not only with regard to T cell infiltration, but also with respect to tumor pathogenesis and biology as well as patient survival and prognosis, the subsequent analyses will focus exclusively on p16^−^ tissues.

### 3.4. Infiltration of T Cells and T Cell Subsets in Tumor Stroma and Tumor Cell Nests

**Figure 3:**
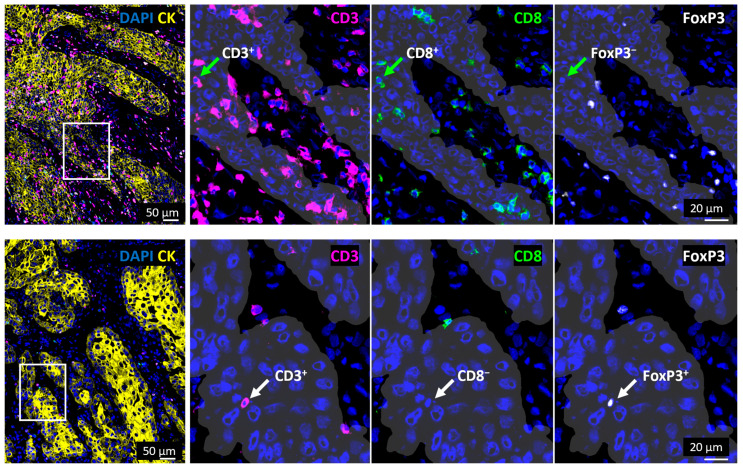
Tumors showing high and low T cell infiltration. Images on the left side show a merged visualization of the spatial distribution of T cell subsets in and outside of tumor cell nests in representative tissue samples with a high (top) or low (bottom) T cell infiltration. Areas marked with white rectangles are shown in a higher magnification on the right side and tumor cell nests are visualized with white shades based on CK staining (yellow). Green arrows (top) mark a CTL (CD3^+^CD8^+^FoxP3^−^) while white arrows (bottom) mark a T_reg_ cell (CD3^+^CD8^−^FOXP3^+^). CK = Cytokeratin, DAPI = 4′,6-Diamidin-2-phenylindol.

Spatial T cell distribution could be assessed accurately based on a CK staining to discriminate between epithelial tumor cell nests and the tumor stroma areas. This revealed an overall 3.7-fold higher T cell density within the stroma (Figure 2C). Moreover, when looking at T cell subtypes, the cell density of each T cell subset was significantly higher in the tumor stroma than in tumor cell nests, demonstrating a similarly increased abundance (CTL 3.5-fold, T_H_ cells 3.5-fold, T_reg_ 2.9-fold). Interestingly, the proportion of T_H_ cells was significantly lower in the tumor cell nests than in the tumor stroma, while the proportion of T_reg_ was significantly higher in the tumor cell nests. Representative images of tumor tissues with high or low T cell infiltration are shown in Figure 3 and Supplementary Figure S2.

### 3.5. T Cell Infiltration and Anatomical Tumor Site

Given the fact that HNSCC originates from several different anatomical sites exhibiting distinct clinical characteristics, we investigated the differences in T cell infiltration according to the localization of the primary tumor (Figure 2D, Supplementary Figure S3). Interestingly, we did not observe significant differences between tumors from various anatomical sites (Supplementary Figure S3).

### 3.6. T Cell Infiltration-Related Cytokine and Chemokine Levels

**Figure 4:**
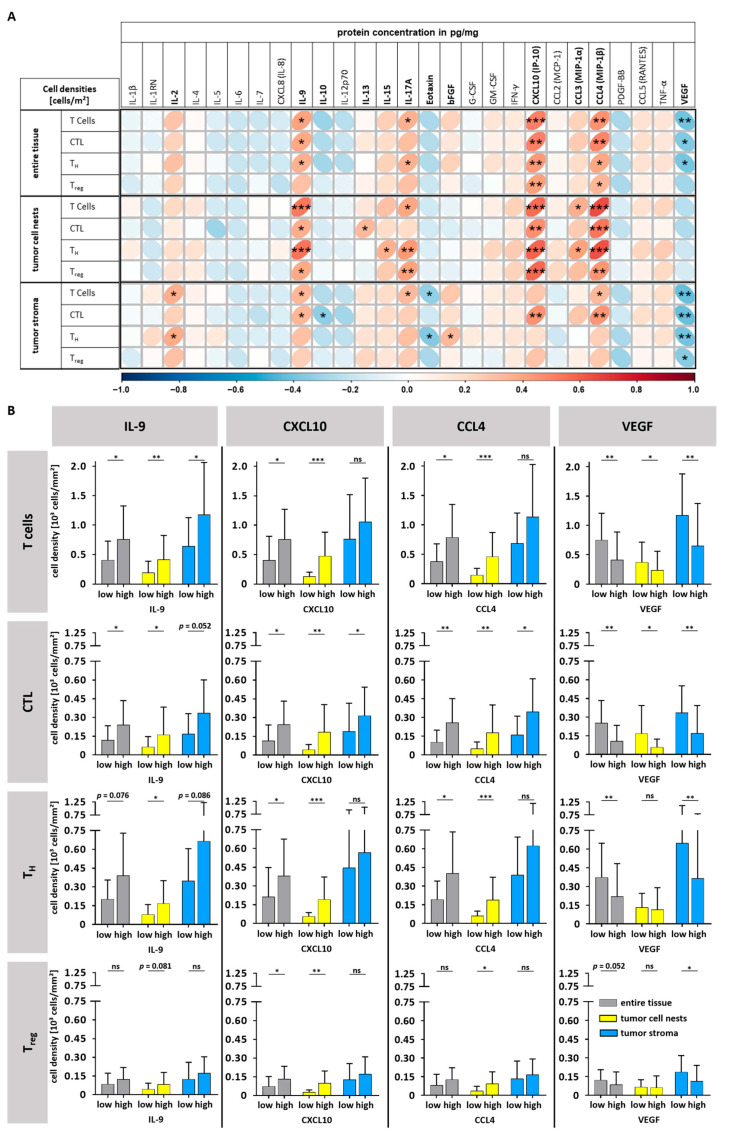
Comparison of T cell densities with cytokine/chemokine levels. (**A**) A correlation chart showing Spearman correlations of T cell densities [cells/mm^2^] (entire tissue, tumor cell nests, and tumor stroma) and corresponding cytokine/chemokine levels. (**B**) T cell densities (general T cells, CTL, T_H_ cells, and T_reg_) dependent on high or low levels of IL-9, CXCL10, CCL4, and VEGF (median cutoff, bars show mean and standard deviation, Mann–Whitney *U* Test). ns = not significant; * *p* < 0.05; ** *p* < 0.01; *** *p* < 0.001, bFGF = basic fibroblast growth factor, CCL = chemokine (C-C motif) ligand, CTL = cytotoxic T cell, CXCL = C-X-C motif chemokine ligand, G-CSF = granulocyte colony-stimulating factor, GM-CSF = granulocyte macrophage colony-stimulating factor, IFN-γ = interferon gamma, IL = interleukin, PDGF-BB = platelet-derived growth factor subunit BB, TC = T cell, T_H_ cell = T helper cell, T_reg_ = regulatory T cell, TNFα = tumor necrosis factor alpha, VEGF = vascular endothelial growth factor.

Next, the concentrations of 27 immune-related cytokines and chemokines were quantified using a multiplex Luminex assay in corresponding HNSCC tissues. An association of protein levels with T cell densities was examined with respect to T cell infiltration in whole tumor tissue as well as in tumor cell nests and tumor stroma only. The results are visualized in a correlation plot (Figure 4A). Altogether, we identified significant correlations for the chemoattractant C-X-C motif chemokine ligand 10 (CXCL10) as well as the chemokine (C-C motif) ligand 4 (CCL4) with higher densities of T cells in general and of CTLs, almost regardless of the tumor compartment analyzed (entire tumor, tumor cell nests or tumor stroma). In addition, the association of CXCL10 and CCL4 levels with the densities of all T cell subtypes increased when the tumor cell nests were specifically analyzed.

Furthermore, the levels of Interleukin-9 (IL-9) correlated positively with the density of general T cells and CTL in the whole tumor tissue as well as in distinct compartments. Again, only in tumor cell nests, an additional association with T_H_ cell and T_reg_ densities was observed.

In contrast, high levels of vascular endothelial growth factor (VEGF) were associated with lower densities of general T cells, CTL, and T_H_ cells in the entire tumor tissue and the tumor stroma. However, as for T_reg_ density, this was restricted to the tumor stroma.

To learn more about how much cytokine and chemokine levels varied in different tumor compartments depending on the T cell infiltration, we grouped the data of the top four proteins, IL-9, CXCL10, CCL4, and VEGF, median-based into high and low. Figure 4B and Supplementary Table S1 illustrate higher levels of IL-9, CXCL10, and CCL4 to be associated with a 1.8- to 2.1-fold higher general T cell infiltration, particularly in tumor cell nests (yellow bars). In contrast, higher VEGF levels were associated with a reduced 1.8-fold lower T cell density particularly in the tumor stroma (blue bars). When looking at the distinct T cell subtypes, we found that CTL infiltration showed the highest fold changes, regardless of the cytokine and the tumor compartment analyzed; for instance, there was a 4.3-fold increase in tumor cell nests in the CXCL10-high group (Figure 4B, Supplementary Table S1).

### 3.7. Survival Analysis

**Figure 5:**
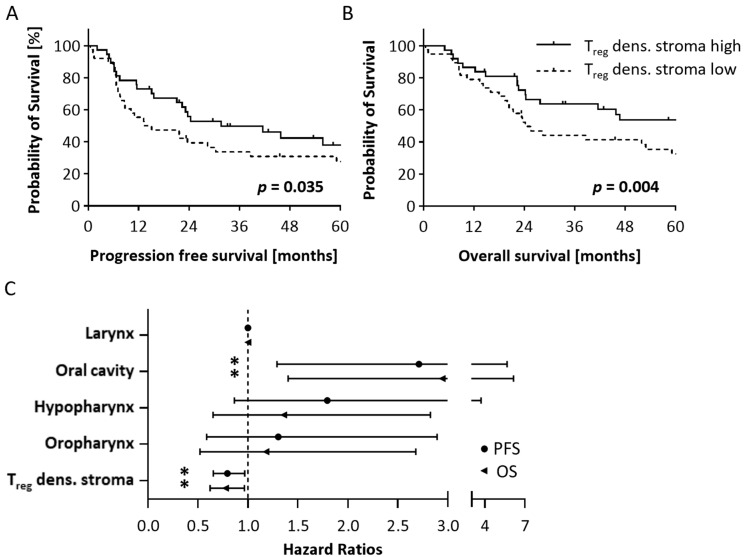
Univariate and multivariate survival analysis. (**A**,**B**) Univariate survival analysis shown as Kaplan–Meier curves demonstrates longer (**A**) PFS and (**B**) OS of patients with high stromal T_reg_ density; *p*-values based on log-rank test (n = 76). (**C**) Forest plot of multivariate analysis using Cox PH regression confirms worse outcome of oral cavity tumors and association of higher stromal T_reg_ densities and longer survival (PFS and OS (n = 76)). Whisker = lower and higher 95% confidence interval, symbols = hazard ratio. * *p* < 0.05, OS = Overall survival, PFS = Progression-free survival, dens. = density, T_reg_ = regulatory T cell.

We finally assessed the impact of T cell infiltration on patient survival. The mean OS of the whole study cohort of patients suffering from p16^−^ HNSCC was 45.8 months and mean PFS was 23.2 months. Except for localization, no other clinical parameter had a significant impact on patient outcome (Supplementary Table S2, Supplementary Figures S4 and S5). With regard to a possible influence of T cell infiltration on survival, we considered the density of different T cell subtypes and their spatial distribution (tumor cell nests vs. stroma) as well as cell ratios of T cell subsets (CTL/T_reg_ ratio and T_H_/CTL ratio). While univariate survival analysis based on a Cox PH model did not reveal any significant associations of general T cells, CTL and T_H_ cells, we observed a significant association with improved OS for the density of T_reg_ infiltration in the tumor stroma (Supplementary Figure S6). Regarding the stromal CTL/T_reg_-ratio, an opposite trend was observed (Supplementary Figure S6). We used Kaplan–Meier curves to visualize the impact of T_reg_ density on survival, splitting the study sample into high and low T_reg_ groups. This confirmed the enhanced OS at high T_reg_ densities and the improved PFS, while no differences for other clinical parameters were observed (Figure 5A,B, Supplementary Tables S3 and S4). Cytokine levels were not associated with patient survival (Supplementary Figure S7).

Subsequent multivariate analyses not only confirmed the prognostic impact of tumor localization but also substantiated stromal T_reg_ densities as an independent prognostic covariate (Figure 5C). Accordingly, a higher stromal T_reg_ cell density was associated with both better PFS (HR = 0.80, *p* = 0.023) and OS (HR = 0.78, *p* = 0.024) (Supplementary Table S5). Thus, the T_reg_ infiltration might serve as a new biomarker for the prognosis of patients suffering from HPV^−^ HNSCC.

## 4. Discussion

In the current study, we analyzed the extent and spatial distribution of tumor infiltrating T cells, cytokine levels, and survival associations in a cohort of 84 HNSCC patients. Seventy-six samples (90%) were p16^neg^, including a well-balanced number of tumors from all major anatomical sites. A robust semi-automated detection method was used to quantify T cells on whole tissue slides on a single cell level. This allowed us to identify an increased stromal T_reg_ cell infiltration in p16^neg^ HNSCC as an independent prognostic factor for better patient survival. Furthermore, we found higher levels of CXCL10, IL-9, and CCL4 in cases with higher numbers of T cells in direct contact with tumor cells, and thus their potential targets, while in these cases, VEGF levels were reduced. In general, a higher T cell infiltration was observed in the tumor stroma as compared to tumor cells nests, but this was significantly lower in a small test sample of p16^+^ and thus presumably HPV^+^ tumors. The impact of the tumor localization on T cell infiltration seemed to be negligible.

One of the major findings of this study is the prognostic relevance of stromal T_reg_ infiltration in p16^−^ HNSCC. A higher density of T_reg_ cells in tumor stroma is associated with better PFS as well as a better OS. Although due to the assumed function of T_reg_ at a first glance, this was unexpected, other authors describe similar findings about survival benefits based on T_reg_ infiltration [23,24,25,26]. However, some other previous studies did not report such a correlation [27,28,29,30]. Reasons for this might be that not all of these studies considered the HPV status of the tumors and some used a less precise quantification method for T cells, such as single-marker immunohistochemical staining with subsequent scoring based on T cell counting in few microscopic fields. A convincing explanation in favor of our findings has been proposed by Ladoire et al. based on the mucosal origin of some cancer entities, including HNSCC [31]. HNSCCs, under physiological conditions, are colonized by over 500 different bacterial species [32]. This results in a substantial infiltration by immune cells such as T cells, macrophages, and neutrophils, even in healthy tissue [33]. This creates an immunological microenvironment primarily aimed at defending against potential pathogens. However, these immune cells produce cytokines, growth factors, and angiogenic proteins, which can favor tumor progression in the case of malignant transformation of the tissue [34,35]. According to this hypothesis, the association of improved survival with higher T_reg_ infiltration might be based on the suppression of an aberrant immune response [36]. This hypothesis has been corroborated by mouse studies, where T_reg_ reduced pathological inflammation driven by immune cells [37]. Additionally, human FasL^+^ T_reg_ can eliminate monocytes and macrophages, thereby reducing the tumor-promoting and immunosuppressive effects of these cells [38]. Moreover, better survival with higher T_reg_ infiltration has also been observed in patients with colorectal cancer [39,40,41]. In line with this observation, the colon mucosa is also persistently colonized by bacteria under physiological conditions, as is the mucosa of the upper aerodigestive tract [31]. Therefore, our study may provide support to resolve inconsistent findings reported the literature. Altogether, there is a strong rationale that increased T_reg_ infiltration in p16^−^ HNSCC can be considered as a marker for a beneficial tumor microenvironment, resulting in a better patient survival, and thus may serve as a new biomarker in this tumor entity.

In contrast to the prognostic value of T_reg_ in the tumor stroma, we found no survival association for T_reg_ infiltration in direct contact with tumor cells. This result aligns with previous studies [24,29,42,43]. However, we cannot exclude the possibility that this observation was biased by our sample size of 76 p16^−^ HNSCC cases, because other studies have suggested a positive survival correlation of T_reg_ infiltration in tumor cell nests based on studies with larger patient cohorts including more than 140 tumor samples [44,45,46]. In order to evaluate whether our study sample was underpowered with regard to a prognostic value of T_reg_ with direct contact to tumor cells, additional analyses on a larger study sample of HPV- HNSCC with a spatial resolution may help to resolve these inconsistent observations. An alternative explanation for why stromal—but not intraepithelial—T_reg_ density predicts outcome is the “immune-excluded” tumor phenotype: regulatory T cells accumulate in the stromal compartment yet fail to penetrate the epithelial tumor nests [47,48]. Such spatial restriction may be driven by stromal remodeling, disrupted chemokine gradients, or TGF-β–mediated exclusion. Paradoxically, a high density of stromal T_regs_ could signal a highly organized, immunologically active microenvironment—even if effector T cells cannot access tumor cells directly. These findings emphasize that prognostic assessments should consider not only the number of infiltrating immune cells but also their precise localization within tumor versus stromal regions.

As a further strength of our study, we analyzed cytokine levels in a considerable subset of the same tumor tissues to learn more about the prerequisites in the tumor niche that allow for an increased T cell infiltration. Higher levels of CXCL10, IL-9, and CCL4 in the tumor tissues were found to be associated with higher numbers of T cells in direct contact with tumor cells. These findings suggest that these cytokines may be relevant to allow a deeper T cell infiltration and thus to reach the cancer cells as potential targets for the cytotoxic activity of tumor-infiltrating T cells. Complementing our protein-level data, Arora et al. recently analyzed HNSCC tumor samples by spatial transcriptomics and demonstrated a significant enrichment of CD8^+^ T cells (adjusted *p* < 0.01) at the tumor’s leading edge, coincident with elevated CXCL10 mRNA expression in these regions compared to the tumor core [49]. This spatial gene-expression pattern mirrors our observation that CXCL10-high tumors harbor the highest fold-change in CTL density within tumor cell nests. Moreover, Shinn et al. have now provided direct functional evidence in murine models that increasing intratumoral CXCL10 levels drives T cell infiltration, further supporting a causal role for this chemokine [50]. In addition, in a complementary stromal context, Li et al. identified a subset of cancer-associated fibroblasts as a key source of CXCL10, demonstrating that these fibroblasts guide T cells into the tumor microenvironment [51]. In line with these spatial and mechanistic insights, CXCL10-high tumors are significantly more infiltrated by all T cell subtypes, but with the highest fold-change for CTL density within tumor cell nests. These findings are of major importance because many new anti-cancer therapies aim for an efficient immune response against cancer cells and therefore it is necessary that T cells be in contact with tumor cells to exert cytotoxic effects. Accordingly, there is a need to find biomarkers of an effective immune cell infiltration, not only into the tumor stroma, to guide therapy decisions. CXCL10 is also called interferon gamma-induced protein 10 (IP-10) as it is secreted in response to IFN-γ by various cell types, including monocytes, endothelial cells, and fibroblasts. It promotes anti-tumor activity and T cell migration into inflammatory tissues [52]. In HNSCC, the CXCR3/CXCL10 axis plays an essential role in the regulation of peripheral blood mononuclear cell chemotaxis [53]. Furthermore, CXCL10 is overexpressed in HNSCC in comparison to normal tissue, and high expression levels of CXCL10 were shown to be associated with better OS in HNSCC patients [54]. Based on these findings, the suitability of CXCL10 to serve as a novel biomarker for effective T cell infiltration in p16^−^ HNSCC should be further studied.

Similarly, we found higher levels of IL-9 in the tumor tissues to be associated with higher T cell infiltration. For instance, tumors that are IL-9 high have 2.1-fold greater densities of CTL in contact with tumor cells than IL-9 low tumors. Originally, IL-9 has been described to be involved in autoimmune diseases, allergic reactions, and parasitic infections. It is known to be a growth factor of T cells and is secreted by multiple cell types including Th9 cells, type 2 innate lymphoid cells, and mast cells [55,56]. In tumor immunology IL-9 is considered a “double-edged sword” because its pleiotropic effects can lead to both pro- and anti-tumor effects [57]. In solid tumors such as breast cancer and melanoma, the anti-tumor role is more pronounced [58,59] whereas in hematological neoplasms such as diffuse large B lymphoma, the lymphocyte growth function can lead to tumor progression [60]. In good agreement with our data, it has been described that IL-9 can be produced by CD8^+^ T cells and that IL-9-producing CD8^+^ T cells convey an enhanced anti-tumor immunity [61,62]. Whether increased numbers of CTL next to tumor cells in IL-9-high p16^−^ HNSCC also indicate an increased anti-tumor immune response and thus might serve as a biomarker deserves further investigation.

Consistent with our observation of a negative correlation between T cells and VEGF, there is evidence that VEGF contributes to immunosuppression by blocking dendritic cell maturation, upregulating PD-L1 on antigen presenting cells, and reducing T cell trafficking [63]. On the other hand, CLL4 has been described to recruit T cells and DCs that form immune aggregates, which is generally associated with a favorable outcome [64]. It has also been previously described to be associated with high CTL infiltration in HNSCC, although this study did not differentiate between stroma and tumor areas [65].

Another interesting finding in our study has been the observation that p16^+^ HNSCCs are more heavily infiltrated by T cells than p16^−^ HNSCCs. Although the small number of p16^+^ tumors can be regarded as a major limitation, our findings are consistent with previous studies [25,29,45,66,67,68]. In addition, our analyses revealed that the increased T cell infiltration in p16^+^ tissues is due to higher absolute densities of CTLs and T_H_ cells within tumor cell nests. However, the proportion of these T cell subtypes relative to the total T cell count does not differ by p16 status. In contrast, the proportion of T_reg_ is lower in p16^+^ tissues, while the T_reg_ density does not change. This suggests that the differences in T cell subtype compositions based on p16 status are driven by a higher infiltration of CTLs and T_H_ cells in p16^+^ HNSCC. This may be attributed to the HPV-associated development of these tumors, in which viral antigens function as unique neoantigens that can be recognized by the patient’s immune system. In virus-associated tumors, a T cell-mediated immune response is particularly effective. For example, studies on HPV^+^ cervical dysplasia have shown that higher T cell infiltration correlates with dysplasia regression [69]. Additionally, gene expression analyses of dysplastic cervical mucosa before and after HPV vaccination revealed increased expression of genes associated with T effector cell phenotype, polarization, function, and activation in the stroma of post-vaccination samples [70]. Therefore, tumor-infiltrating lymphocytes play a crucial role in tumor immunity in HPV^+^ tumor tissues, indicating the relevance of T cell infiltration as a prognostic parameter. Although our study sample of HPV^+^ tumors was too small to allow a survival analysis, other studies have shown that increased tumor cell nest T cell infiltration of HPV^+^ tumors is indeed associated with improved patient prognosis [29,68,71]. In contrast, infiltration by CD4^+^ cells in HPV-associated tumors does not appear to correlate with patient survival [29,67]. Overall, we conclude that consideration of the p16 status is essential in T cell infiltration studies of HNSCC to avoid an unwanted bias due to the substantially higher T cell infiltration of p16^+^ tumors.

Similarly, we found marked spatial differences regarding T cell infiltration of the tumor stroma as compared to tumor cell nests. Specifically, the tumor stroma of p16^−^ HNSCC is approximately 3.5-fold more highly infiltrated than the tumor cell nests. Other authors have also shown a higher infiltration of the tumor stroma [23,29,72,73]. However, unlike previous studies, we evaluated HNSCCs from all four major tumor sites and conducted a comprehensive subclassification of T cells into CTLs, T_H_ cells, and T_reg_. Thus, we were able to show that the increased infiltration of the tumor stroma applies to all major anatomical sites and to all T cell subtypes. This is of particular importance because immune checkpoint blockade has already become a relevant treatment option for patients suffering from HNSCC and has been shown to not only increase the number of tumor-infiltrating lymphocytes but also to alter their spatial distribution within the tumor microenvironment [74,75]. Checkpoint inhibition—especially via anti-PD-1 antibodies—has been associated with a transition from an immune-excluded to a more inflamed phenotype, characterized by the migration of clonally expanded CD8^+^ T cells from the stromal compartment into tumor epithelial regions [76]. As for HNSCC, the relevance of spatial T cell dynamics is underscored by recent findings from the KEYNOTE-689 study [77]. While detailed immunophenotyping data have not yet been published, the study demonstrated a significant improvement in event-free survival with perioperative pembrolizumab compared to standard therapy. These results raise the possibility that checkpoint inhibition may induce not only systemic immune activation but also qualitative changes within the tumor microenvironment, potentially enabling T cells to infiltrate and exert effector functions within previously excluded epithelial compartments. Taken together, these findings highlight the therapeutic potential of modulating the spatial distribution of T cells through checkpoint blockade and support the notion that spatial immune architecture should be considered as a key parameter in evaluating and optimizing immunotherapeutic strategies in HNSCC.

Interestingly, we found no differences in T cell infiltration between various anatomical tumor sites of p16^−^ HNSCC. While some authors have reported similar findings [23,78], others describe opposing results [46,66,79]. In particular, oropharyngeal tumors were described to be more heavily infiltrated by T cells. As mentioned earlier, these controversial observations may be due to differences in the study design and methodological approach used, including p16 status, and software-based T cell quantification. However, we cannot exclude the possibility that a small subpopulation of CD3^+^CD8^−^ may have been misclassified through the indirect assessment of CD4^+^ cells.

## 5. Conclusions

Our concise analyses on the spatial distribution of different T cell subsets and their association with cytokine levels in 84 well-characterized HNSCC tissues revealed that stromal T_reg_ infiltration in p16^−^ HNSCC could serve as an independent prognostic marker for improved patient survival, while higher CXCL10, IL-9, and CCL4 levels might be useful biomarkers to predict a deeper infiltration of T cells into tumor cell nests and thus to reach their potential target cells. Our data strongly suggest that, in future studies investigating T cell infiltration in HNSCC, it is important to consider the p16 status, as well as the spatial distribution of T cells to avoid an unwanted bias and to prognostically exploit the spatial information.

## Figures and Tables

**Table 1:** Patient characteristics of the staining cohort.

		T Cell Infiltration		Cytokine Levels	
		*n* = 84	[%]	*n* = 40	[%]
**Sex**	Male	69	82.1	33	82.5
	Female	15	17.9	7	17.5
**Age [years]**	Median [Min.–Max.]	60.1 [30–85]		58.4 [30–78]	
**Tumor site**	Oral cavity	20	23.8	14	35.0
	Oropharynx	22	26.2	8	20.0
	Larynx	22	26.2	10	25.0
	Hypopharynx	20	23.8	8	20.0
**T stage**	T1	8	9.5	1	2.5
	T2	31	36.9	15	37.5
	T3	21	25.0	12	30.0
	T4	24	28.6	12	30.0
**N stage**	N0	19	22.6	11	27.5
	N1/N2	65	77.4	29	72.5
**UICC stage**	I/II	6	7.1	4	10.0
	III/IV	78	92.9	36	90.0
**Postoperative** **therapy**	None	16	19.0	11	27.5
	Radiation alone	36	42.9	16	40.0
	CRT	32	38.1	13	32.5
**p16 status**	Positive	8	9.5	-	-
	Negative	76	90.5	40	100

CRT = chemoradiotherapy, Min. = Minimum, Max. = Maximum, UICC = Union Internationale Contre le Cancer.

## Data Availability

The original contributions presented in this study are included in the article/Supplementary Materials. Further inquiries can be directed to the corresponding author.

## Supplementary Materials

The following supporting information can be downloaded at: https://www.mdpi.com/article/10.3390/cells14110789/s1, Figure S1: From cytokeratin staining to distance transformation in StrataQuest; Figure S2: Tumors showing high and low T cell infiltration; Figure S3: T cell infiltration based on the anatomical site; Figure S4: Univariate survival analysis based on clinical characteristics; Figure S5: Survival analysis based on the anatomical tumor site; Figure S6: Univariate survival analysis based on T cell infiltration parameters; Figure S7: Survival analysis based on cytokine levels; Table S1: Fold-changes of mean T cell densities based on IL-9, CXCL10, CCL4 or VEGF concentrations; Table S2: Univariate survival analysis of patient characteristics; Table S3: Patient characteristics of the T_reg_ survival cohort; Table S4: Univariate survival analysis of T cell infiltration parameters; Table S5: Multivariate survival analysis of patient characteristics.
