# Supplementary material for: Spatial Distribution and Prognostic Value of T Cell Subtypes and Immune Biomarkers in p16-Negative HNSCC
Source: Cells. 2025 May 27;14(11):789. doi: 10.3390/cells14110789 (PMC12153745; doi:10.3390/cells14110789)
Supplement: Supplementary file 1 [file cells-14-00789-s001.zip › cells-3610829-supplementary.pdf]

Supplementary Materials

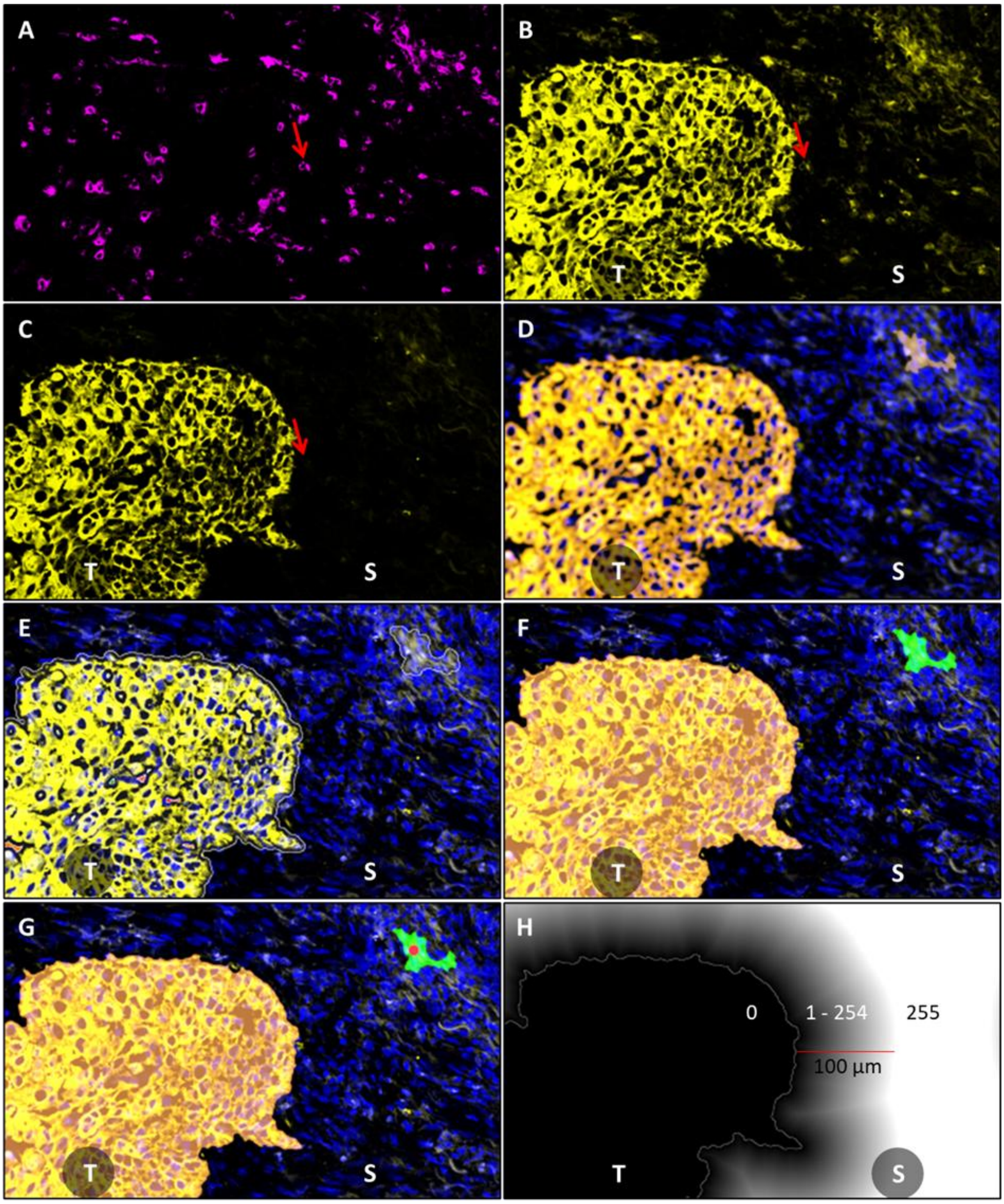

Figure S1. From cytokeratin staining to distance transformation in StrataQuest. A| The CD3 signal from the AF647 channel was also detected in a diminished intensity in the **B**| cytokeratin (CK) channel. **C**| By subtracting the two images, the corrected CK image was obtained. **D**| After automatic detection of a preliminary tumor area based on the CK staining, **E**| holes caused by negative staining of tumor cell nuclei were identified automatically and **F**| added to the tumor region. **G**| Through manual correction, areas with unspecific CK staining were marked (red dot) and removed. **H**| The final tumor region served as the basis for a distance transformation. The epithelial tumor cell nests were assigned to a grayscale value of 0, while the tumor stroma was

assigned to grayscale values ranging from 1 to 255, depending on their distance from the tumor cell nests. CK = cytokeratin, T = tumor cell nest, S = tumor stroma, red arrows indicate a T cell.

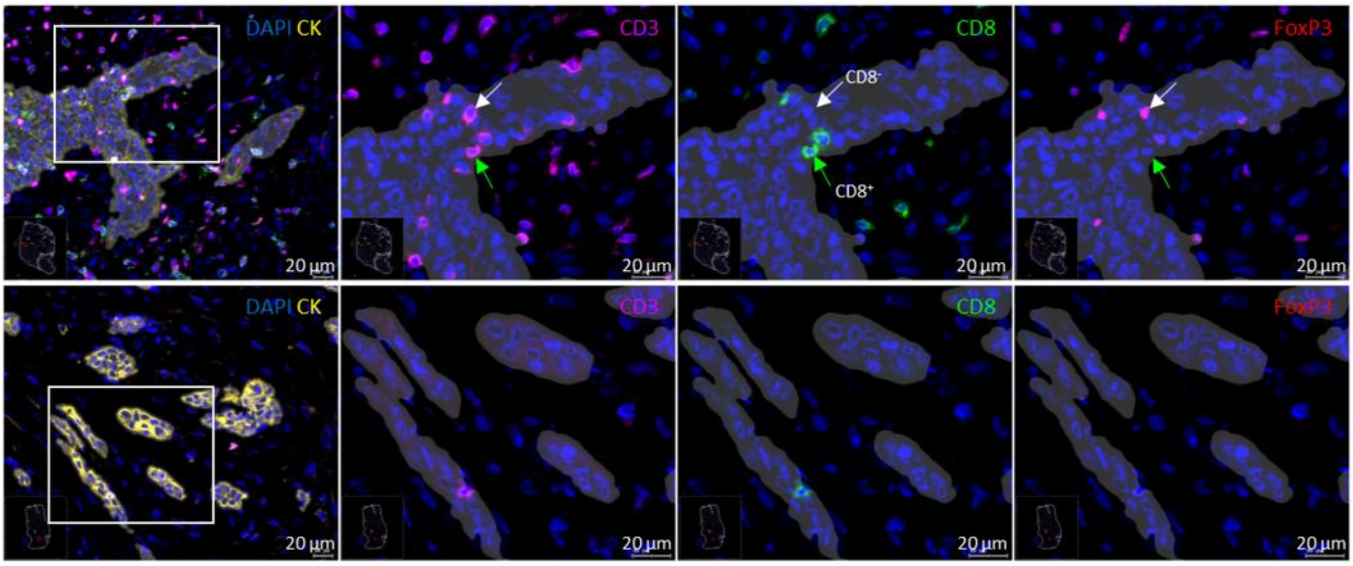

Figure S2. Tumors showing high and low T cell infiltration. Left: merged visualization of the spatial distribution of T cell subsets in and outside of tumor cell nests in representative tissue samples with a high (top) or low (bottom) T cell infiltration. Areas marked with white rectangles are shown in a higher magnification in the other three panels. Tumor cell nests are visualized with white shades based on CK staining (yellow). Green arrows (top) mark a CTL ($CD3^+CD8^+FoxP3^-$) while white arrows (bottom) mark a T_{reg} cell ($CD3^+CD8^-FOXP3^+$). CK = Cytokeratin, DAPI = 4′, 6-Diamidin-2-phenylindol.

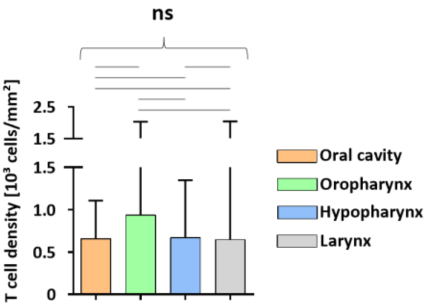

Figure S3. T cell infiltration based on the anatomical site. T cell density in the entire tumor tissue of $p16^-$ HNSCCs based on the anatomical site. Statistical tests for the comparisons were Kruskal Wallis Test and uncorrected Dunn's Test. ns = not significant

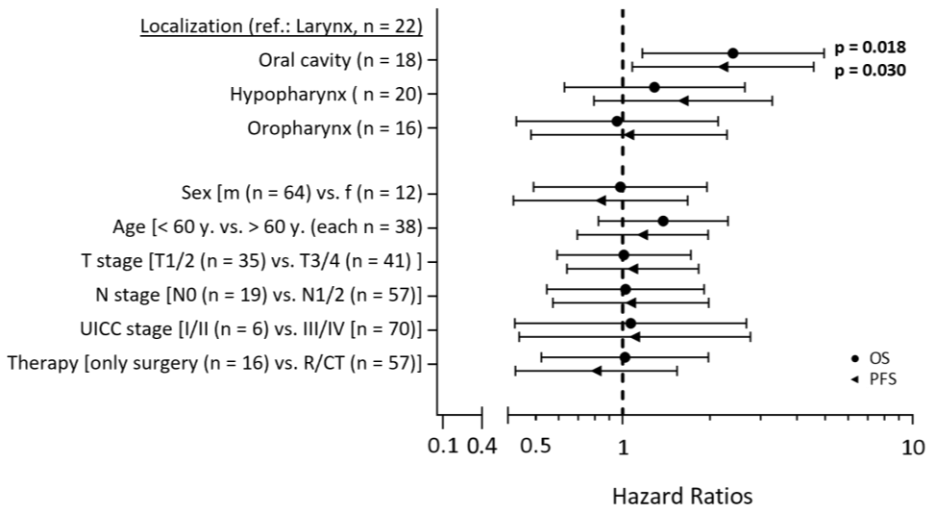

Figure S4. Univariate survival analysis based on clinical characteristics. Hazard ratios of overall survival (dots) and progression-free survival (triangles) with 95 % confidence interval resulting from univariate Cox PH regression are shown (n = 76). Only p16-negative HNSCC patients were included. Patients suffering from larynx carcinoma had a significant better survival compared to patients with oral cavity carcinoma (OS: HR = 2.40, p = 0.018; PFS: HR = 2.22, p = 0.030). n = number, R/CT = radio and/or chemotherapy, ref. = reference, y = year.

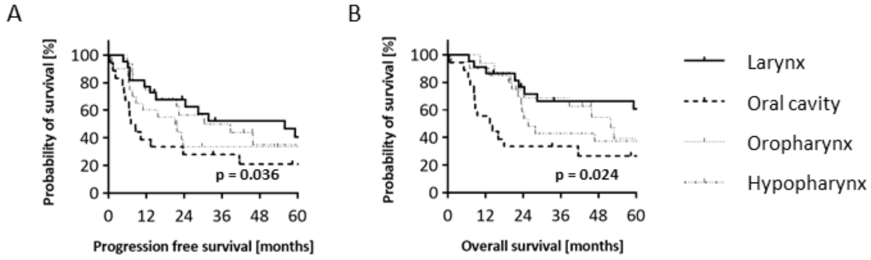

Figure S5. Survival analysis based on the anatomical tumor site. Kaplan-Meier curves visualize observed survival differences between patients with tumors of different anatomical sites (n = 76). **A**| Progression-free (PFS) **B**| Overall survival (OS) (p-value for larynx vs. oral cavity)..

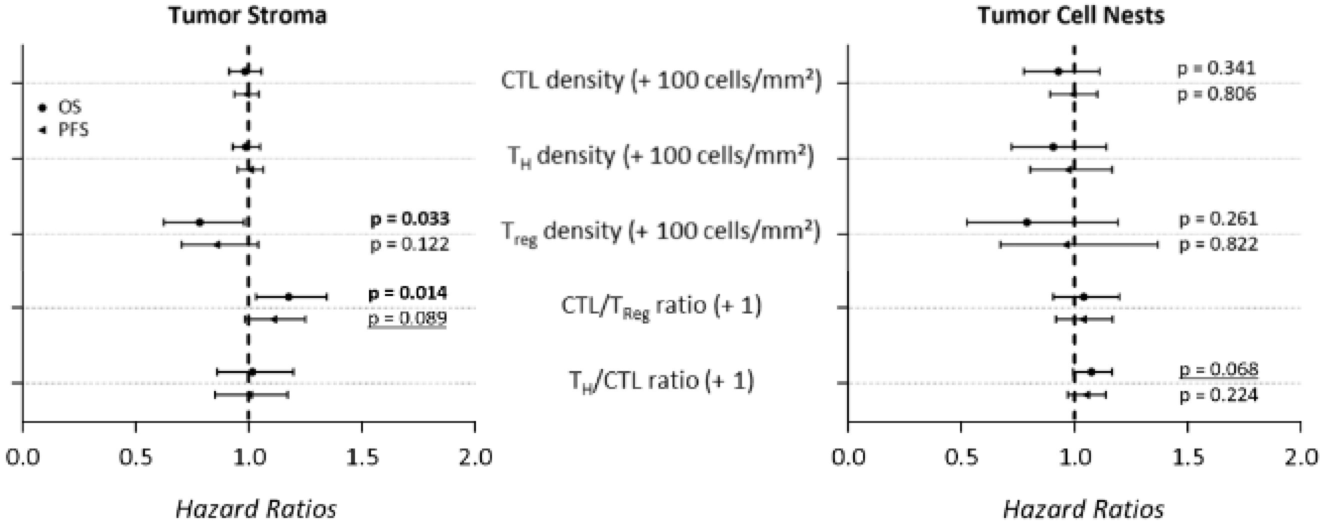

Figure S6. Univariate survival analysis based on T cell infiltration parameters. Hazard ratios of overall survival (dots) and progression-free survival (triangles) with 95 % confidence interval resulting from univariate Cox PH regression are shown. Only patients with p16-negative HNSCC were included (n = 76). The univariate Cox PH regression revealed significant associations with patient survival for all parameters related to T_{reg} cells in the tumor stroma. The univariate analysis of T cell infiltration in tumor cell nests did not reveal significant associations with either overall survival (OS) or progression-free survival (PFS). CTL = cytotoxic T cell, T_H = T helper cell, T_{reg} = regulatory T cell.

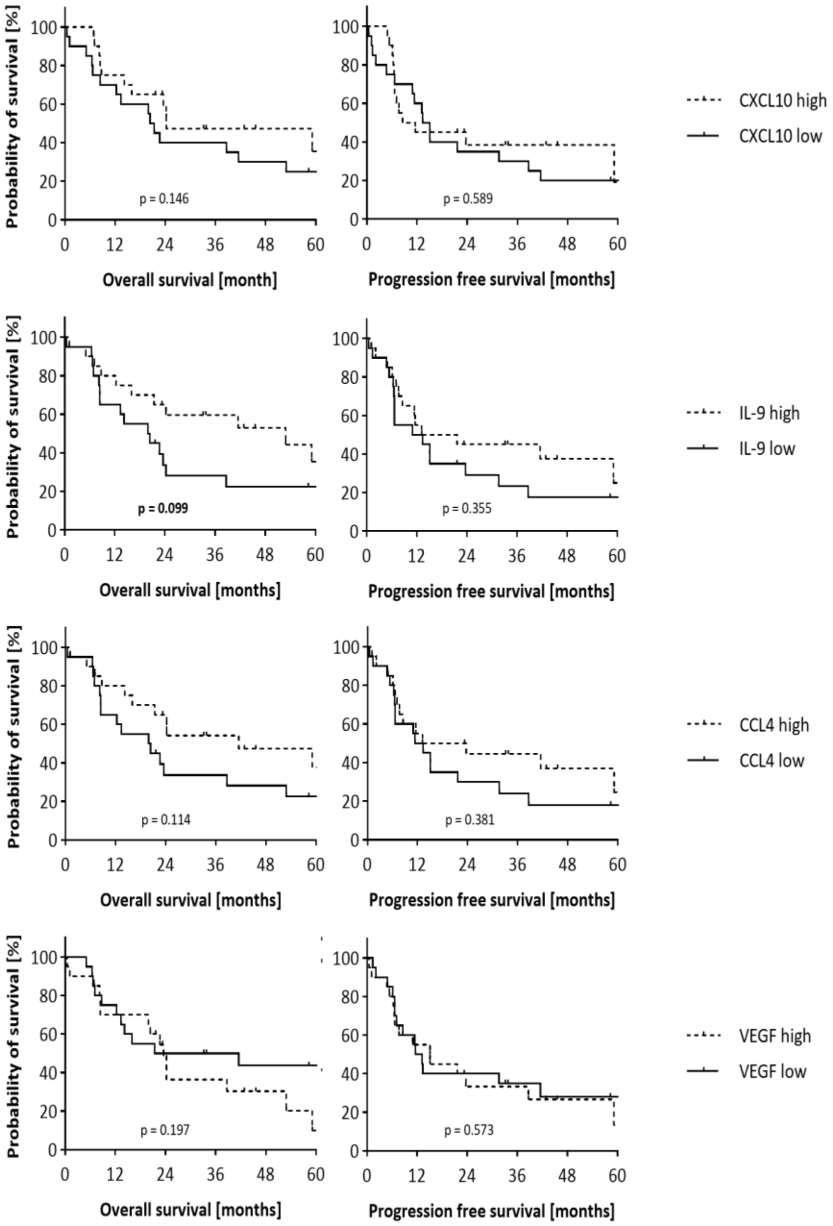

Figure S7: Cytokine level-associated survival. OS (left) and PFS (right) of patients depending on high or low levels of CXCL10, IL-9, CCL4 or VEGF, respectively. p value resulting from log-rank test.

Table S1. Fold-changes of mean T cell densities based on IL-9, CXCL10, CCL4 or VEGF concentrations.

		IL-9			CXCL10			CCL4			VEGF		
		**ET**	**TCN**	**TS**	**ET**	**TCN**	**TS**	**ET**	**TCN**	**TS**	**ET**	**TCN**	**TS**
**TC**	**FC**	1.9	**2.2**	1.8	1.9	3.7	1.4	2.1	**3.2**	1.7	**1.8**	1.6	**1.8**
	p	*	**	*	**	***	ns	*	***	ns	**	*	**
**CTL**	**FC**	2.1	**2.5**	2.0	2.2	4.3	1.7	2.5	**3.5**	2.2	2.4	**2.9**	2.0
	p	*	*	0.052	*	**	*	**	**	*	**	*	**
**T_H**	**FC**	1.9	**2.1**	1.9	1.8	3.4	1.3	2.1	**3.2**	1.6	1.7	1.2	**1.8**
	p	0.076	*	0.086	*	***	ns	*	***	ns	**	ns	**
**T_{reg}**	**FC**	1.5	1.9	1.4	1.8	3.6	1.3	1,6	**2.6**	1.2	1.4	1.1	**1.7**
	p	ns	0.081	ns	*	**	ns	0.081	*	ns	0.052	ns	*

The cytokine assay study collective (n = 40) was split into two groups (low vs. high) based on median cytokine concentrations. Fold-changes of mean cell densities of general T cells, cytotoxic T cells, T helper cells, and regulatory T cells between low and high groups were calculated. Highest fold-changes of significant results are bold (statistical test: Mann-Whitney *U* test). CTL = cytotoxic T cell, ET = entire tissue, FC = fold-change, TCN = tumor cell nest, T_H = Helper T cell, T_{reg} = regulatory T cell, TS = tumor stroma, * $p < 0.05$; ** $p < 0.01$; *** $p < 0.001$, ns not significant

Table S2. Univariate Survival Analysis of Patient Characteristics.

			PFS				OS			
		**n**	**HR**	**95%-CI**		**p**	**HR**	**95%-CI**		**p**
**Localization**	Larynx	22	1				1			
	Oral cavity	18	2.216	1.078	4.555	**0.031**	2.404	1.168	4.950	**0.017**
	Hypopharynx	20	1.616	0.796	3.280	0.184	1.287	0.628	2.635	0.491
	Oropharynx	16	1.050	0.482	2.289	0.902	0.956	0.429	2.131	0.912
**Sex**	m	64	1				1			
	f	12	0.838	0.419	1.676	0.617	0.981	0.492	1.956	0.957
**Age**	< 59.4 y.	38	1				1			
	> 59.4 y.	38	1.171	0.696	1.971	0.551	1.261	0.741	2.147	0.392
**T stage**	1/2	35	1				1			
	3/4	41	1.084	0.641	1.831	0.764	1.010	0.594	1.719	0.970
**N stage**	0	19	1				1			
	1/2	57	1.066	0.573	1.983	0.839	1.022	0.547	1.910	0.946
**UICC-stage**	I/II	6	1				1			
	III/IV	70	1.100	0.438	2.761	0.839	1.066	0.424	2.675	0.892
**Therapy**	Surgery only	16	1				1			
	Postop. Ther.	57	0.817	0.431	1.549	0.536	1.036	0.535	2.008	0.916

CI = Confidence Interval, f = female, HR = Hazard Ratio, m = male, n = number, OS = overall survival, PFS = progression-free survival, postop. Ther. = postoperative Therapy, y = years, p < 0.05 highlighted in green.

Table S3. Patient Characteristics of the T_{reg} survival cohort.

		T_{reg} density							
		Tumor stroma				Tumor cell nest			
		high		low		high		low	
		n = 37	[%]	n = 38	[%]	n = 38	[%]	n = 38	[%]
**Sex**	Male	31	83.8	32	84.2	31	81.6	33	86.8
	Female	6	16.2	6	15.8	7	18.4	5	13.2
**Age [years]**	Median [Min.–Max.]	58.5 [42–75]		60.1 [30–78]		59.8 [42–76]		58.6 [30–78]	
**Tumor site**	Oral cavity	8	21.6	10	26.3	11	28.9	7	18.4
	Oropharynx	8	21.6	8	21.1	7	18.4	9	23.7
	Larynx	11	29.7	11	28.9	10	26.3	12	31.6
	Hypopharynx	10	27.0	9	23.7	10	26.3	10	26.3
**T stage**	T1	5	13.5	3	7.9	5	13.2	3	7.9
	T2	12	32.4	14	36.8	13	34.2	14	36.8
	T3	10	27.0	7	18.4	10	26.3	7	18.4
	T4	10	27.0	14	36.8	10	26.3	14	36.8
**N stage**	N0	9	24.3	10	26.3	8	21.1	11	28.9
	N1/ N2	28	75.7	28	73.7	30	78.9	27	71.1
**UICC stage**	I/II	3	8.1	3	7.9	3	7.9	3	7.9
	III/IV	34	91.9	35	92.1	35	92.1	35	92.1
**Postoperative therapy**	None	7	18.9	9	23.7	8	21.1	8	21.1
	Radiation alone	15	40.5	16	42.1	17	44.7	14	36.8
	CRT	15	40.5	13	34.2	12	31.6	14	36.8

n = 75 for the tumor stroma and n = 76 for the tumor cell nests because one tumor tissue showed no tumor stroma areas. CRT = chemoradiotherapy, Min. = Minimum, Max. = Maximum

Table S4. Univariate Survival Analysis of T cell infiltration parameters.

		PFS				OS			
**Tumor stroma**	**Interval**	**HR**	**95-%-CI**		**p**	**HR**	**95-%-CI**		**p**
**TC density**	+500 cells/mm²	0.964	0.864	1.076	0.515	0,969	0,856	1,096	0,618
**CTL density**	+100 cells/mm²	0.980	0.924	1.040	0.507	0.987	0.925	1.054	0.699
**T_H density**	+100 cells/mm²	0.997	0.967	1.029	0.873	0.998	0.964	1.032	0.897
**T_{reg} density**	+100 cells/mm²	0.820	0.669	1.005	0.056	0.794	0.632	0.998	**0.048**
**CTL proportion**	+10 %	1.013	0.812	1.266	0.906	1.056	0.838	1.332	0.643
**T_H proportion**	+10 %	1.176	0.944	1.466	0.149	1.173	0.935	1.472	0.168
**T_{reg} proportion**	+10 %	0.762	0.570	1.019	0.067	0.743	0.557	0.992	**0.044**
**CTL/T_{reg} ratio**	+1	1.064	0.952	1.191	0.275	1.117	0.989	1.260	0.074
**T_H/CTL ratio**	+1	1.024	0.899	1.167	0.722	1.027	0.892	1.182	0.713
**Tumor cell nests**	**Interval**	**HR**	**95-%-CI**		**p**	**HR**	**95-%-CI**		**p**
**TC density**	+500 cells/mm²	0.961	0.699	1.321	0.806	0.807	0.522	1.248	0.336
**CTL density**	+100 cells/mm²	0.991	0.892	1.102	0.873	0.929	0.776	1.112	0.423
**T_H density**	+100 cells/mm²	0.970	0.806	1.167	0.745	0.908	0.722	1.141	0.406
**T_{reg} density**	+100 cells/mm²	0.960	0.673	1.369	0.822	0.791	0.525	1.192	0.263
**CTL propotion**	+10 %	1.030	0.859	1.235	0.749	0.969	0.806	1.166	0.739
**T_H proportion**	+10 %	0.980	0.824	1.166	0.820	1.067	0.891	1.276	0.482
**T_{reg} proportion**	+10 %	0.984	0.757	1.281	0.907	0.936	0.727	1.204	0.605
**CTL/T_{reg} ratio**	+1	1.037	0.919	1.171	0.553	1.047	0.912	1.203	0.515
**T_H/CTL ratio**	+1	1.059	0.961	1.168	0.248	1.083	0.985	1.190	0.101

n = 75 for the tumor stroma and n = 76 for the tumor cell nests because one tumor tissue showed no tumor stroma areas. CI = confidence interval, CTL = cytotoxic T cell, HR = Hazard ratio, n = number, TC = T cell, T_H = T helper cell, T_{reg} = regulatory T cell, $p < 0.1$ highlighted in yellow, $p < 0.05$ highlighted in green.

Table S5. Multivariate Survival Analysis of Patient Characteristics.

			PFS				OS			
		**n**	**HR**	**95%-CI**		**P**	**HR**	**95%-CI**		**p**
**Localization**	Larynx	22	1				1			
	Oral cavity	18	2.713	1.293	5.692	**0.008**	2.944	1.404	6.170	**0.004**
	Hypopharynx	20	1.795	0.865	3.721	0.116	1.359	0.653	2.827	0.412
	Oropharynx	16	1.304	0.587	2.895	0.515	1.183	0.522	2.680	0.688
**T_{reg} dens. stroma**	+ 100 cells/mm²	76	0.797	0.655	0.969	**0.023**	0.776	0.623	0.967	**0.024**

CI = Confidence Interval, dens. = density, HR = Hazard Ratio, n = number, OS = overall survival, PFS = progression-free survival, p < 0.05 highlighted in green.
